# The strategic debriefing. Incorporating strategic dialogue in the standard debriefing after the scenario

**DOI:** 10.15694/mep.2020.000210.1

**Published:** 2020-09-28

**Authors:** Giorgio Capogna, Emanuele Capogna, Giorgio Nardone

**Affiliations:** 1 EESOA Simulation Center; 2 Strategic Therapy Center

**Keywords:** Debriefing, Strategic dialogue, Strategic Therapy, Medical Education.

## Abstract

This article was migrated. The article was marked as recommended.

The most common debriefing structure usually includes at least three phases, a time to confront reactions and/or emotions felt during the scenario, an analysis of events and a summary or application phase, in which the learning acquired throughout the debriefing is solidified and the major take- home messages condensed. However, using only open-ended questions and/or the puls/delta method, participants’ attitudes may remain unchanged and result in only superficial reflections of the scenario. The purpose of this article is to describe and propose incorporating strategic dialogue after the healthcare high fidelity scenario in the standard debriefing.

Strategic dialogue alternates analogical and digital language and uses metaphors and paraphrases, guiding the participant to live a corrective emotional experience, the primary cause of the change. The structure of the strategic dialogue is based on the use of questions with the illusion of alternatives, restructuring paraphrases, the evocation of sensations and summary redefinitions, in order to reach the discovery that leads to change.

The simulation method is well suited and integrated with strategic communication. Both aim at change, which must first be experienced and then explained.

The central focus of strategic short therapy is the corrective emotional experience in which the patient modifies his or her vision of reality through concrete emotional experiences. The change, in order to be rapid and effective, must primarily produce a real personal experience of transformation on a perceptive-emotional level and only then can it be the subject of cognitive reasoning. The change must first pass from the experience phase and only then to the level of cognitive awareness.

The strategic language, an effective tool in strategic psychotherapy and business problem solving, is ideal and complementary to the standard debriefing methods, making them more performing and functional because, next to common logic, it makes use of non-ordinary logical language.

## Background

The debriefing after the scenario is an essential component of simulation-based learning and is defined as an interactive, bidirectional, and reflective discussion or conversation involving some level of facilitation to assist the reflective process. Several debriefing approaches exist, and their common denominator is the facilitative approach to be adopted by the specifically trained instructors ( Sawyer et al., 2016; Kolb and Kolb, 2009; Gaba et al., 2015; INACSL 2016; Der Sahakina et al., 2015).

The most common debriefing structure usually includes at least three phases, a time to confront reactions and/or emotions felt during the scenario, an analysis of events and a summary or application phase, in which the learning acquired throughout the debriefing is solidified and the major take- home messages condensed ( Sawyer et al., 2016).

Our simulation center has developed a model for debriefing after simulation, built on the strategical dialogue, ( Nardone and Salvini, 2007; Watzlawick, Beavin and Jackson, 1967; Nardone and Watzlawick 1999; Watzlawick and Nardone, 1997; Nardone and Portelli, 2005; Nardone and Watzlawick, 2005; Nardone and Balbi, 2015) to enrich the standard, commonly used, debriefing procedure.

The strategic model deals with the way man perceives and manages his own reality through communication with himself, others and the world, transforming it from dysfunctional to functional, in order to operate on it. The strategic dialogue, alternating analogical and digital language, using metaphors and paraphrases, leads the participant to live a corrective emotional experience, the primary cause of the change. The structure of the strategic dialogue is based on the use of questions with the illusion of alternatives, restructuring paraphrases, evocation of sensations and summary redefinitions, in order to reach the discovery that leads to change ( Nardone and Salvini, 2007).

In this paper we describe the “strategic debriefing”, which, while maintaining the phases of the traditional, well established debriefing, is enriched by a deeper and more persistent investigation whose efficacy is confirmed, in the application phase, in the participant’s discovery of concrete and feasible objectives, agents of clinical and behavioral change in the field.

## 1. Reaction Phase

Almost all the debriefings include a time to confront reactions and/or emotions felt during the scenario. Here the debriefer explores the participants’ reactions to the scenario and the emotional impact of the simulation experience, thus enabling participants to ‘vent’ before completing the rest of the debriefing ( Sawyer et al., 2016).

The strategic dialogue can help to better highlight the emotions perceived immediately after the scenario. In the reaction phase, with our proposed strategic approach, the participant acquires the inner awareness of the real basic sensation/deep emotion that the scenario has aroused in him: by evoking it, recognizing it and becoming aware of it, he can let it flow and, with the help of the strategic debriefer, use it in a constructive sense.

The exploration of feelings and emotions is done in a more structured way with questions like: “Can you tell me concisely what you are feeling right now? Does this feeling resonate with you? Are there situations in your life or in your daily life that activate this same feeling? Which ones? Is it the same or different from the previous scenario(s)? If different, how?” The debriefer uses the paraphrase technique (see the analytical phase) to summarize synthetically (i.e: “correct me if I’m wrong, you told me that you feel xxx and that you usually live this same feeling in xy situations...”) and concludes with: “Which of the four basic emotions, fear, pleasure, anger, pain, do you refer this feeling to?” or, alternatively, he hypothesizes and proposes to the participant the basic emotion that he was feeling during or immediately after the scenario.

As in real life, even during the simulated scenario, fear is the basic emotion that occurs most frequently, although it is often superficially reported and masked, for example, by anxiety (performance), prejudice (I do something because I am afraid that something else will happen to me or to the patient), and/or insecurity (fear of making mistakes). In the face of fear one is paralyzed and often amazed at the speed with which it establishes itself and conditions our actions insidiously, without our permission. The debriefer, through strategic dialogue, can make the participant observe how fear should be considered as a resource and not a limit. Fear is in fact a mechanism that, within physiological limits, is positive and, indeed, helps to overcome and resolve stressful situations. It is only when it exceeds a certain threshold, becoming panic or a chronic dysfunctional reaction, can it have negative consequences on performance.

It is therefore not a question of repressing or inhibiting the emotional response, but of regulating it by managing the way we perceive what triggers it. In this way the participant acquires the inner awareness of the real sensation/basic emotion that the scenario has aroused in him: by evoking it, recognizing it and being aware of it, he can let it flow and better leave his assigned scenario role, better face the debriefing and consider it as a cognitive learning tool.

Awareness of emotions is a pivotal point in change and being able to verbalize it provides access to adaptive information and the trend of action of each emotion. Making contact with his own “feeling” he is able to connect more easily to his needs (in this case learning) and is motivated to satisfy them. “There is nothing in the intellect that is not in the senses before” (Thomas Aquinas): a good reaction phase opens the way for a good debriefing.

## 2. Descriptive Phase

Similar to the descriptive phase of a standard debriefing, the strategic debriefing also explores, in the same way, what happened during the scenario through the participants’ eyes and accounts at this stage ( Sawyer et al., 2016; Rudolph et al., 2006). The description process leads the group through an “agreed-upon description” immediately after the scenario, action by action, limiting the discussion to facts and avoiding emotions.

However, in the strategic briefing we are proposing, at the end of the descriptive phase, that the debriefer uses the paraphrase technique (see the analytical phase) to summarize what occurred non- judgmentally, by establishing the group’s agreement with what happened, was experienced and perceived by individuals and the group itself.

## 3. Analytical Phase

As in the typical debriefing, the questions are initially general and broad, and then narrow down depending on the participant’s answers, concentrating on particular characteristics of the situation, highlighting the critical points ( Rudolph et al., 2006). For example, the beginning of the conversation could highlight key elements through self-assessment, using the “plus/delta” method, where the facilitator asks open-ended questions like “what went well?, what’s the best thing you did? (plus) and “what could be changed?, what would you do differently?”. (delta). ( Smith-Jentsch et al., 2008). Alternatively, or complementarily, the debriefer can start with open “Who, Where, How, When, What” questions. However, using only open-ended questions and/or the puls/delta method, participants’ attitudes may not change in depth.

For this reason we use the above mentioned types of conversation as a preliminary bridge to the strategic dialogue, that not only deepens the causes of the observed results but also promotes a change in participants’ attitudes. For example, in a leadership standard debriefing the first question asked could be: “Was there a leader?” and then “Who?”. After this first, typical open-ended question the “supposed leader” is asked: “Did you feel like a leader?” so exploring the participant’s feelings, preparing him to reflect on his actions. After his eventual confirmation, the debriefer usually goes deeper by asking the motivations of his feeling, i.e. what he did, verbal and non-verbal, that made him understand that he was the leader and enlarging the discussion with the team (“What behavior made you aware that he was the leader?”). At this point we introduce some tools of the strategic dialogue such as a series of closed questions with the illusion of alternatives, which are structured questions with only two possible answers. The interlocutor can then choose which of the two is best suited to his case.

These types of questions are not simply instrument of knowledge, but also of intervention in the direction of change, provoking in the pupil new ways of feeling and reacting to his reality which was previously trapped in his perceptions and sometimes dysfunctional ( Nardone and Salvini, 2007; Watzlawick, Beavin and Jackson, 1967).

A typical illusion of alternative question could be: “In your opinion should the leader concentrate on doing the therapy, maybe losing sight of the general picture, or should he remain outside the group’s action, to better coordinate the group itself?” In this case the participant, through this further illusion of answer question made by the debriefer during the scenario, receives a further perceptual reinforcement of the correctness of his previously recognized discovery, that it is better for the leader to remain outside the group to better coordinate.

Before proceeding further the debriefer will then use the paraphrase tool ( Nardone and Salvini, 2007; Watzlawick, Beavin and Jackson, 1967; Nardone and Watzlawick 1999; Watzlawick and Nardone, 1997; Nardone and Portelli, 2005; Nardone and Watzlawick, 2005; Nardone and Balbi, 2015) that confirms that the reflection is going in the right direction and allows the perception of the participants to be anchored to the new perspective of the scenario.

For example: “Correct me if I’m wrong, XX organized and coordinated the work, stopped the group from time to time with a 10x10 reassessment of the clinical situation, did not take an active part in the practical activities and was able to listen to his collaborators, all typical actions of a leader”. Saying “correct me if I’m wrong” makes the participant feel that he is leading the process of discovery dialogue, thus making him feel gratified rather than disqualified. This restitution by the debriefer of the participant’ discoveries in the form of paraphrases also serves to consolidate the discoveries, highlighting how they came about from their own input and not imposed a priori by the debriefer, which in turn reaffirms his role as “facilitator” and not “teacher”.

Blaise Pascal’s statement “The same words in different sequences produce different results” well fits the restructuring paraphrase methodology, the maneuver the debriefer performs every time he manages to define a problem with each of the participants after asking open-ended questions, and then, under the illusion of answering, obtains the definition of the problem that occurred during the scenario. No evaluation or interpretation is proposed, but rather, modestly (“correct me if I’m wrong, if I understand correctly”... etc.) and therefore non-judgmentally, a verification of the learning process and understanding of the problem that has occurred is requested, while obtaining an openness to the new perspectives and solutions proposed by the participant himself and/or his teammates. The paraphrase creates a collaborative relationship between debriefer and learner and bypasses any misunderstandings and resistance. The participant feels accepted and put in the first person, the author of the discovery concerning the problem presented and its resolution.

It also helps the participant to consolidate the conviction that even if mistakes have been made, the most important thing is what you do with those mistakes because, quoting Aldous Huxley, “reality is not what happens to us, but what we do with what happens to us”.

After the first series of questions, we begin to go deeper, “funnel-shaped”, into the issue of leadership, delving into its aspects and how they were conducted during the scenario. After a series of open questions, possibly followed by the illusion of answer questions, the debriefer can paraphrase again to find the agreement of the group on what has been analyzed. If the material is sufficient, the debriefer can this time use a restructuring paraphrase associated with an evocative sentence. The technique consists of using the participant’s words to reformulate the definition of the problem but this time using rhetorical figures that fit the participant’s argument and feeling to facilitate the change, because “before convincing the intellect, it is necessary to touch and prepare the heart” (Blaise Pascal). In the case of the debriefing on leadership, we could summarize as follows: “we have therefore seen that XX was a good leader, in fact he took the situation in hand, delegated appropriately, asked his chief and accepted the proposals of his collaborators. However, he could have been more careful when the patient’s condition suddenly changed. But thanks to the anesthetist’s intervention and the suggestions of the nurse who reported the monitor parameters in good time, there were no problems. On the contrary, thanks to teamwork the patient improved quickly! Even a good orchestra conductor can sometimes misunderstand, but if he conducts real masters, the orchestra can cover the misunderstanding”. The feeling you want to evoke is that the team is made up of professionals (the orchestra masters) and not just simple collaborators or wingmen (nurses or other subordinate doctors) who can represent a barrier to possible mistakes or oversights of a seemingly perfect leader.

The evocative language uses all the rhetorical figures and poetic forms: aphorisms, metaphors, anecdotes, concrete examples, narratives or counter-senses. This technique is used to create aversion to attitudes or behaviors that need to be interrupted or changed and lead in an exhilarating way towards those reactions that need to be stimulated or increased.

For example, a nurse frustrated by the apparent futility of his or her marginal role during a scenario of high specialist complexity may recognize (and discover) at the end of the strategic questions posed by the debriefer that his or her suggestions have produced improved therapeutic outcomes. The debriefer, instead of stopping to note the participant’s discovery, could use persuasive language to reinforce the positive attitude that could be decisive in real practice. He could say: “Knowing how to propose yourself can sometimes help those (the leader) who are in a position where they cannot see or hear. A good team member is sometimes the eye and ear of the leader...”.

## 4. Application Phase

Almost all the debriefing methods include a final phase, in which the learning acquired throughout the debriefing is solidified and the major take-home messages condensed ( Sawyer et al., 2016).

In our model we added the concept, borrowed from strategic problem solving, of defining the objective. In concrete terms, the participant is asked not only to share what he has learned, but is also asked to reflect on how the debriefing experience can be the first step for a personal change that can in turn bring about a positive change in clinical hospital activity. To do this, the definition of the SMART (Specific, Measurable, Achievable, Relevant, Time-bound) objective ( Doran, 1981) is introduced: Specific, i.e. concrete and clear, Measurable, i.e. defined in terms of observable results, Achievable, based on constraints and resources, Relevant, i.e. realistic, reasonable and time-bound, i.e. achievable in a given period of time. The debriefer then asks the question so that the “I take home” answers the realization of a SMART objective, and in particular, is the smallest feasible step of concrete change. The participant’s definition of an objective that is SMART is the result of a good strategic debriefing. For example, usually, after a traditional debriefing, to the questions: “What have you learned?, What are you taking home?” the participant answers: “that it is important to communicate using the closed circuit method”. After a strategic debriefing the answer is usually more concrete: “at the first emergency, I will ask the nurse for feedback on the action, calling him by name and looking him in the eyes”.

At the end of the application phase, the debriefer can again use evocative language, quickly redefining for each participant or group the objective of change using an “echo effect” closure that leaves a suggestion, using a short sentence (aphorism, story, anecdote, quotation), that works as an anchor and an emotional stabilization of the discovery made by the participants.

## Discussion

Strategic communication allows the debriefer to be more effective in high fidelity simulation. The simulation method is well suited and integrated with strategic communication. Both aim at change, which must first be experienced and then explained.

The central focus of strategic short therapy is the corrective emotional experience in which the patient modifies his or her vision of reality through concrete emotional experiences. Equally, whoever participates in a strategic debriefing after a simulated scenario, lives a corrective emotional experience and is helped to break his perceptual patterns, triggering the premises for a real long term change in his clinical behavior.

The change, in order to be rapid and effective, must primarily produce a real personal experience of transformation on a perceptive-emotional level and only then can it be the subject of cognitive reasoning. The change must first pass from the experience phase and only then to the level of cognitive awareness.

Even if it is well recognized that by understanding the non-ordinary logic of a problem (which is often based on the logic of belief, paradox and contradiction), we can come to choose the best strategies to bring about effective change ( Watzlawick, Beavin and Jackson, 1967; Nardone and Watzlawick, 1999), our report has some limitations. We are aware that our hypothesis is based on observational experience, and its validation by objective measurement is needed. While as yet, no study has looked at the specific outcomes of this strategic intervention in simulation, the huge thirty years clinical experience with strategic therapy in the field of psychology ( Nardone and Salvini, 2007; Watzlawick, Beavin and Jackson, 1967; Nardone and Watzlawick, 1999; Watzlawick and Nardone, 1997; Nardone and Portelli, 2005; Nardone and Watzlawick 2005; Nardone and Balbi, 2015) and our recent post course feedbacks are very encouraging. A quantitative validation is anyway expected in due course.

In addition, our center usually trains expert physicians rather than medical students and trainees, who may need different ways of debriefing, according to different learning goals, and therefore we feel that the strategic modifications to the debriefing may still need to be verified with young doctors.

## Conclusion

In conclusion, strategic language, a well-established and effective tool in strategic psychotherapy and business problem solving ( Nardone and Salvini, 2007; Watzlawick, Beavin and Jackson, 1967; Nardone and Watzlawick, 1999; Watzlawick and Nardone,1997; Nardone and Portelli, 2005; Nardone and Watzlawick, 2005; Nardone and Balbi, 2015) may be used as an additional tool to any of the standard debriefing methods, which may become more performing and functional because, next to common logic, the strategic dialogue makes use also of the non-ordinary logical language.

## Take Home Messages

• We have introduced the strategic dialogue in the debriefing after high fidelity simulation scenario.
• The strategic dialogue alternates analogical and digital language, uses metaphors and paraphrases, leading the participant to live a corrective emotional experience, which is the primary cause of the change.
• The strategic language is ideal and complementary to the standard debriefing methods, making them more performing and functional.

## Notes On Contributors

### Giorgio Capogna, MD

Giorgio Capogna graduated in Medicine at the University of Rome in 1978 and specialized in Anesthesiology and Intensive Care in 1981. He was the OB Anesthesia Director at FBF Hospital and Head of the Anesthesia Dept at CdR Hospital in Rome, Italy. He is Director of the European School of Obstetric Anesthesia and of EESOA Simulation Center, Rome, Italy. ORCID ID: https://orcid.org/0000-0003-2298-8905

### Emanuele Capogna

Emanuele Capogna received his master degree in strategic problem solving and strategic communication in 2019. He is debriefer and simulation specialist at EESOA Simulation Center, Roma, Italy. ORCID ID: https://orcid.org/0000-0002-4053-1274

### Giorgio Nardone, PhD

Giorgio Nardone graduated at the University of Siena, Faculty of Education in 1981 and has a MSci degree in Psychology. He is founder and Director of the Strategic Therapy Centre of Arezzo, a Research, Training and Psychotherapy Institute, which gave the start to the modern evolution of Brief Strategic Therapy.
