# Supplementary material for: The strategic debriefing. Incorporating strategic dialogue in the standard debriefing after the scenario
Source: MedEdPublish (2016). 2020 Sep 28;9:210. [Version 1] doi: 10.15694/mep.2020.000210.1 (PMC10699390; doi:10.15694/mep.2020.000210.1)
Supplement: Peer review history

# Reviewer report: Reviewer response for version 1

**DOI:** 10.21956/mep.20131.r31072

**Published:** 2020-12-12

**Authors:** Megan Anakin

**Affiliations:** 1 University of Otago

This review has been migrated. The reviewer awarded 3 stars out of 5 As a member of a simulation interest group at my medical school, I am intrigued by articles that we can discuss at our meetings to inform our practice. I appreciate that the senior author has been publishing about therapeutic interviewing techniques for over two decades and brings these ideas to health professional educators. The strategic debriefing technique is described in detail.To enhance the use of this article by health professional educators interested in improving their debriefing practice during simulation scenarios, please consider presenting evaluation data or other research evidence from your simulation centre to support the claim at the end of the background section that the strategic debriefing will provide “a deeper and more persistent investigation whose efficacy is confirmed, in the application phase, in the participant's discovery of concrete and feasible objectives, agents of clinical and behavioural change in the field”. In the discussion section, please consider explaining how health professional educators might apply the strategic debriefing technique in different educational contexts, such as with undergraduate students and with experienced practitioners in workplace settings. In both these cases, there may be a time pressure to complete the debriefing phase of the simulation scenario. Please describe how long the strategic debriefing might take and explain how it might be modified to use in busy time pressured workplace learning environments.

Reviewer Expertise:

NA

No decision status is available

# Reviewer report: Reviewer response for version 1

**DOI:** 10.21956/mep.20131.r31071

**Published:** 2020-11-16

**Authors:** Pier Luigi Ingrassia

This review has been migrated. The reviewer awarded 4 stars out of 5 The paper addresses the debriefing after the scenario being an essential component of simulation-based learning.I enjoyed reading the paper: the manuscript is comprehensive, easy to read and well managed, although some limitations are present in the study. The main objective of this paper is to describe a new model for debriefing after simulation, built on the strategical dialogue. The presented debriefing maintains the structure of the traditional debriefing but is characterized by a deeper investigation to drive participants to discovery easy objectives to achieve and identify agents of clinical and behavioral change. To do so, the authors present vary techniques, such as the use of questions with the illusion of alternatives, restructuring paraphrases, evocation of sensations or summary redefinitions which can help participants learn from the simulated experience.I agree this technique will be useful to those working in simulation-based education and it may become complementary to the standard debriefing methods. Nevertheless, the proposed technique lacks of evidence about its effectiveness. And I wish the authors will design research study to assess it.

Reviewer Expertise:

NA

No decision status is available

# Reviewer report: Reviewer response for version 1

**DOI:** 10.21956/mep.20131.r31073

**Published:** 2020-10-12

**Authors:** Mohamed Al-Eraky

**Affiliations:** 1 University of Dammam

This review has been migrated. The reviewer awarded 4 stars out of 5 This is an interesting paper that provides a clear anatomy for debriefing after the scenario in simulated-based learning. I would like to suggest another layer that may go along with the four phases, indicated above. There’s a great potential to teach professionalism and ethics in the phases of Reaction and Analysis. The Descriptive phase is a great opportunity to improve communication skills. While the Application phase is a venue to advance self-directed learning and develop individual professional plans. I hope these reflective thoughts might help authors to capitalise on their model to inspire teachers and clinicians to address relevant domains in clinical practice and medical education. Good luck.

Reviewer Expertise:

NA

No decision status is available
